# Sex differences in outcomes after acute coronary syndrome vary with age: a New Zealand national study

**DOI:** 10.1093/ehjacc/zuad151

**Published:** 2023-12-12

**Authors:** Nikki J Earle, Robert N Doughty, Gerry Devlin, Harvey White, Craig Riddell, Yeunhyang Choi, Andrew J Kerr, Katrina K Poppe

**Affiliations:** Department of Medicine, University of Auckland, Park Avenue, Graton, Auckland 1023, New Zealand; Department of Medicine, University of Auckland, Park Avenue, Graton, Auckland 1023, New Zealand; Cardiology, Te Toka Tumai Auckland Hospital, Auckland, New Zealand; Cardiology, Gisborne Hospital, Gisborne, New Zealand; Cardiology, Te Toka Tumai Auckland Hospital, Auckland, New Zealand; Department of Medicine, University of Auckland, Park Avenue, Graton, Auckland 1023, New Zealand; Section of Epidemiology and Biostatistics, University of Auckland, Auckland, New Zealand; Department of Medicine, University of Auckland, Park Avenue, Graton, Auckland 1023, New Zealand; Section of Epidemiology and Biostatistics, University of Auckland, Auckland, New Zealand; Middlemore Hospital, Counties Manukau District Health Board, Auckland, New Zealand; Department of Medicine, University of Auckland, Park Avenue, Graton, Auckland 1023, New Zealand

**Keywords:** Acute coronary syndromes, Sex differences, Clinical outcomes following acute coronary syndromes

## Abstract

**Aims:**

This study investigated age-specific sex differences in short- and long-term clinical outcomes following hospitalization for a first-time acute coronary syndrome (ACS) in New Zealand (NZ).

**Methods and results:**

Using linked national health datasets, people admitted to hospital for a first-time ACS between January 2010 and December 2016 were included. Analyses were stratified by sex and 10-year age groups. Logistic and Cox regression were used to assess in-hospital death and from discharge the primary outcome of time to first cardiovascular (CV) readmission or death and other secondary outcomes at 30 days and 2 years. Among 63 245 people (mean age 69 years, 40% women), women were older than men at the time of the ACS admission (mean age 73 vs. 66 years), with a higher comorbidity burden. Overall compared with men, women experienced higher rates of unadjusted in-hospital death (10% vs. 7%), 30-day (16% vs. 12%) and 2-year (44% vs. 34%) death, or CV readmission (all *P* < 0.001). Age group-specific analyses showed sex differences in outcomes varied with age, with younger women (<65 years) at higher risk than men and older women (≥85 years) at lower risk than men: unadjusted hazard ratio of 2-year death or CV readmission for women aged 18–44 years = 1.51 [95% confidence interval (CI) 1.21–1.84] and aged ≥85 years = 0.88 (95% CI 0.83–0.93). The increased risk for younger women was no longer significant after multivariable adjustment whereas the increased risk for older men remained.

**Conclusion:**

Men and women admitted with first-time ACS have differing age and comorbidity profiles, resulting in contrasting age-specific sex differences in the risk of adverse outcomes between the youngest and oldest age groups.

## Graphical Abstract

**Graphical Abstract:**
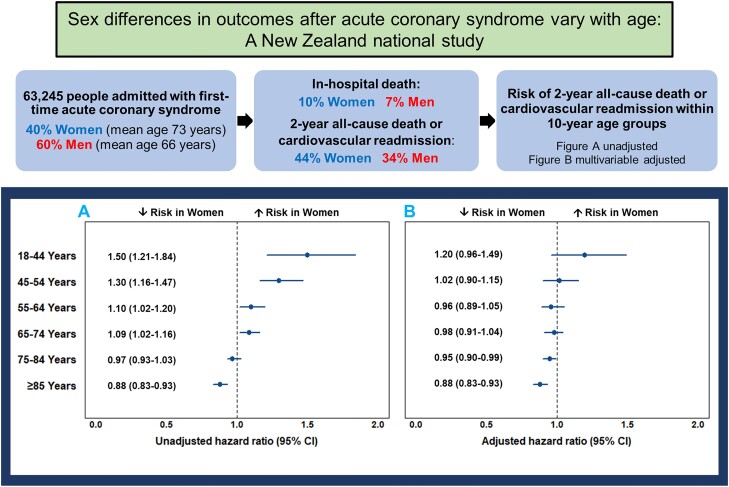

## Introduction

Ischaemic heart disease (IHD) and its manifestation as acute coronary syndrome (ACS) is a major cause of morbidity and mortality for both men and women worldwide.^1^ In New Zealand (NZ), although rates of ACS hospitalizations and IHD mortality are declining,^2,3^ people experiencing an ACS are at increased risk of recurrent events and IHD remains the leading single cause of death.^4,5^

Sex differences in the life-course of IHD are well known, but due to the perception that women are at low risk, heart disease in women has been understudied.^6,7^ Compared with men, women experiencing ACS have a higher comorbidity burden, are less likely to receive guideline-recommended therapies, and have higher rates of in-hospital and long-term mortality and readmissions.^8–13^ While many studies have found these differences in outcomes to be largely explained by age and comorbidities,^8,9^ men and women have different distributions of cardiovascular (CV) risk factors and comorbidities by age,^14^ meaning we should consider age-specific sex differences in ACS outcomes.

The largest difference in clinical outcomes following ACS is seen in younger patients, with several studies identifying younger women to be at higher risk of adverse events than men, especially among those with ST-elevation myocardial infarction (STEMI).^12,13,15–19^

Recent data have shown that while 1-year mortality and CV readmission for ACS are decreasing in NZ, women have a persistently higher unadjusted risk compared with men.^2^ Beyond this, sex differences in outcomes after an ACS admission in NZ have not been investigated. Here, we utilize national datasets to investigate age-specific sex differences in short and long-term clinical outcomes after hospitalization for a first-time ACS.

## Methods

### Study cohort and variables

Every individual in NZ is allocated a unique National Health Identifier (NHI) number, enabling the recording of interactions with the health system. All patients admitted to hospital for a confirmed first-time ACS between 1 January 2010 and 31 December 2016 in the NZ National Hospitalization Dataset were included in this study. The following International Statistical Classification of Diseases and Related Health Problems, Tenth Revision, Australian Modification (ICD10-AM) codes were used to identify ACS admissions (primary and secondary diagnoses): STEMI (I21.0-I21.3, I22.0, I22.1, I22.2, I22.8, and I22.9); non-STEMI (NSTEMI; I21.4); MI unspecified (I21.9 and I25.2); and unstable angina (UA) (I20.0). First-time ACS was defined as having no prior ACS hospitalizations in the National Hospitalization Dataset (with data available from 1988 onwards).

Demographic and clinical characteristics were sourced from national datasets. Where >1 ethnic group was recorded with a patient’s NHI, ethnicity was prioritized in this order: Māori, Pacific peoples, Indian, Chinese/Other Asian, Other (including Middle Eastern, Latin American, African, and Other), and European. The NZDep2013 score (an area-based measure of relative deprivation ranging from 1 to 10; least to most deprived) was used to estimate socio-economic position.^20^ Medical history variables were based on prior hospitalizations. The M3 multimorbidity index is constructed from discharge ICD-10 codes of 55 chronic comorbid conditions in the 5 years prior to the index ACS admission.^21^

### Clinical outcomes

In-hospital death and outcomes at 30 days and 2 years after discharge for the index ACS hospitalization were obtained from the National Hospitalization and Mortality datasets for all patients. The primary outcome was time from discharge to first CV readmission or death from any cause. This included any acute admission with a primary or secondary ICD-10-AM code for MI, UA, other IHD, heart failure, ischaemic or haemorrhagic stroke, transient ischaemic attack, other cerebrovascular diseases, or peripheral vascular disease (PVD). The primary outcome also included unplanned procedures for ischaemic coronary or PVD, as coded using the Australian Classification of Health Interventions system (see Supplementary material online, *Table S1*). Secondary outcomes included first CV readmission, CV death, and all-cause death, with the cause of death defined by ICD-10 codes in the National Mortality dataset.

### Statistical analysis

Key descriptors of the study cohort are presented as mean and standard deviation (SD), median (interquartile range), or frequency (percentage). The study population was stratified by sex and then by sex within six 10-year age groups (18–44, 45–54, 55–64, 65–74, 75–84, and ≥85 years).

Logistic regression was used to model in-hospital death, with multivariable models adjusted for sex, age, M3 multimorbidity index, type of ACS, ethnicity, and NZDep2013 score quintile. Age was removed for models developed by age group.

Cox regression was used to model the time from hospital discharge to the first of all-cause death, CV readmission, or end of follow-up (2 years after discharge), stratified by sex. Multivariable Cox models within age group categories were adjusted for sex, M3 multimorbidity index, type of ACS, ethnicity, and NZDep2013 score quintile. The potential interaction between 10-year age group and sex was tested for in a multivariable model of the whole cohort. Unadjusted and multivariable adjusted models were also performed for each secondary outcome by 10-year age groups.

A *P*-value of <0.05 was deemed statistically significant. Analyses were carried out using RStudio^22,23^ version 2023.03.0 and R packages.

## Results

### Baseline characteristics

Between 2010 and 2016, a total of 63 245 people were admitted to NZ hospitals with a first-time ACS, 25 502 (40%) women and 37 743 men (*Table 1*). The mean age at the time of admission was 69 (SD 14) years, and the ethnic distribution was as follows: 77% European, 11% Māori, 5% Pacific peoples, 3% Indian, 3% Chinese or Other Asian, and 2% others. The ACS diagnosis was 55% NSTEMI, 22% STEMI, 18% UA, and 5% MI unspecified.

**Table 1:** Clinical characteristics of men and women admitted with first-time acute coronary syndrome from 2010 to 2016

	Whole group	Women	Men	*P*
	*n* = 63 245	*n* = 25 502 (40%)	*n* = 37 743	
**Demographics**				
Age, years, mean (SD)	69 (14)	73 (14)	66 (14)	<0.001
Ethnicity, *n* (%)				
European	48 551 (77%)	19 843 (78%)	28 708 (76%)	<0.001
Māori	6645 (11%)	2905 (11%)	3740 (10%)	<0.001
Pacific people	3260 (5%)	1222 (5%)	2038 (5%)	<0.001
Indian	2205 (3%)	620 (2%)	1585 (4%)	<0.001
Chinese or other Asian	1619 (3%)	572 (2%)	1047 (3%)	0.244
Other	965 (2%)	340 (1%)	625 (2%)	0.668
History of atherosclerotic CVD^a^	16 008 (25%)	6917 (27%)	9091 (24%)	<0.001
Prior PCI or CABG	2788 (4%)	740 (3%)	2048 (5%)	<0.001
History of PVD	4245 (7%)	1793 (7%)	2452 (7%)	0.008
History of atrial fibrillation	7174 (11%)	3409 (13%)	3765 (10%)	<0.001
History of heart failure	5439 (9%)	2871 (11%)	2568 (7%)	<0.001
M3 index, mean (SD)	0.39 (0.69)	0.47 (0.73)	0.34 (0.66)	<0.001
NZ deprivation index (quintile)				
1 (least deprived)	9136 (14%)	3256 (13%)	5880 (16%)	<0.001
2	10 902 (17%)	4371 (17%)	6531 (17%)	0.450
3	12 381 (20%)	5040 (20%)	7341 (20%)	0.474
4	14 981 (24%)	6342 (25%)	8639 (23%)	<0.001
5 (most deprived)	15 023 (24%)	6223 (24%)	8800 (23%)	0.004
NZDep missing	822 (1%)	270 (1%)	552 (2%)	<0.001
Type of ACS				
STEMI	13 750 (22%)	4545 (18%)	9235 (25%)	<0.001
NSTEMI	35 058 (55%)	14 912 (59%)	20 146 (53%)	<0.001
Unstable angina	11 045 (18%)	4480 (18%)	6565 (17%)	0.581
MI unspecified	3392 (5%)	1595 (6%)	1797 (5%)	<0.001
**Clinical outcomes**				
In-hospital mortality	5054 (8%)	2441 (10%)	2613 (7%)	<0.001
**30 days** ^b^				
Death or CV readmission	4924 (9%)	2326 (10%)	2598 (7%)	<0.001
CV readmission^c^	3774 (7%)	1769 (8%)	2005 (6%)	<0.001
All-cause death	1495 (3%)	729 (3%)	766 (2%)	<0.001
CV death	781 (1%)	386 (2%)	395 (1%)	<0.001
**2 years** ^b^				
Death or CV readmission	19 232 (33%)	8830 (38%)	10 402 (30%)	<0.001
CV readmission	9173 (16%)	4490 (19%)	4683 (13%)	<0.001
All-cause death	14 558 (25%)	6552 (28%)	8006 (23%)	<0.001
CV death	4312 (7%)	2151 (9%)	2161 (6%)	<0.001

Values are *n* (column percentage), median (interquartile range), or mean (standard deviation).

ACS, acute coronary syndrome; CABG, coronary artery bypass graft; CVD, cardiovascular disease; NSTEMI, non-ST-elevation myocardial infarction; PCI, percutaneous coronary intervention; PVD, peripheral vascular disease; STEMI, ST-elevation myocardial infarction.

^a^Includes admissions for atherosclerotic CVD excluding prior ACS.

^b^30-day, 2-year events exclude in-hospital events.

^c^Includes all CV readmissions not just those within the composite outcome (same with other secondary outcomes).

Women were significantly older than men at the time of the ACS admission [mean 73 (SD 14) vs. 66 years (SD 14)] and were more likely to have a higher overall comorbidity burden as well as previous hospitalizations for atherosclerotic CV disease (CVD), atrial fibrillation, and heart failure (all *P* < 0.001; *Table 1*). The type of ACS also varied by sex, with NSTEMI being more common in women than in men (59% vs. 53%, *P* < 0.001), while STEMI was less common in women (18% vs. 25%, *P* < 0.001).

### Clinical outcomes

A total of 5054 (8%) people died during the index ACS hospitalization, 2441 (10%) women and 2613 (7%) men (*P* < 0.001). Women had a higher unadjusted risk for in-hospital death [odds ratio (OR) for women 1.42; 95% confidence interval (CI) 1.34–1.51, *P* < 0.001] (see Supplementary material online, *Table S2).* After multivariable adjustment for age, M3 multimorbidity index, type of ACS, ethnicity, and NZ deprivation index, the OR for women compared with men was 0.94 (95% CI 0.88–1.01, *P* = 0.064) (see Supplementary material online, *Table S3*).

**Figure 1:**
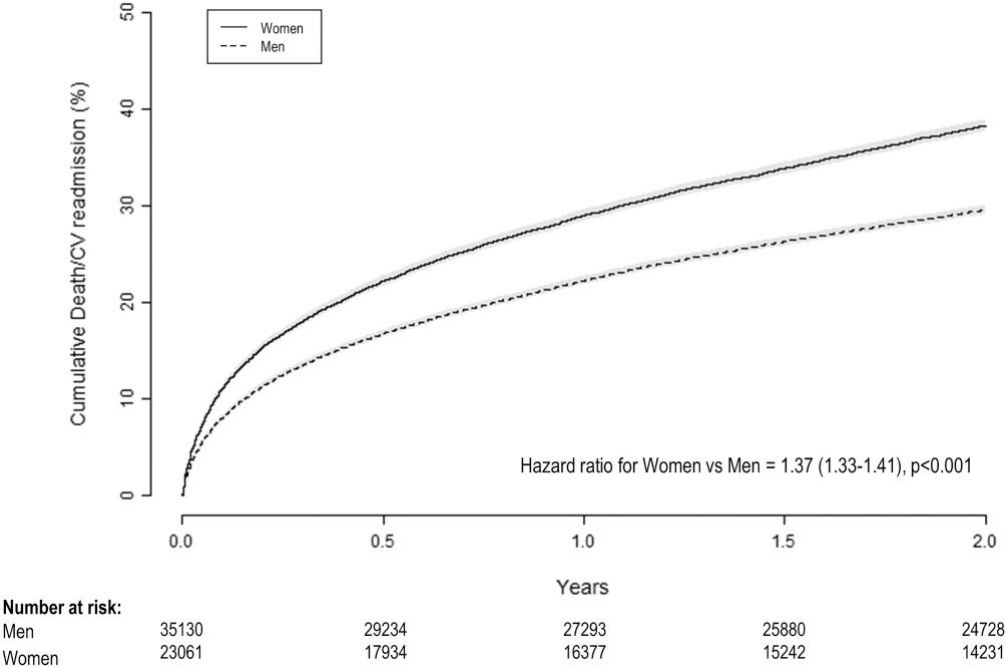
Unadjusted cumulative event curves for women and men after discharge from a first-time acute coronary syndrome admission. Shading represents 95% confidence intervals. CV, cardiovascular.

Of the 58 191 people alive at discharge, 8514 (13%) either died or had a CV readmission within 30 days, with women being at a higher risk compared with men (16% vs. 12%, *P* < 0.001). At 2 years, 38% of people had experienced death or CV readmission, 44% of women and 34% of men, *P* < 0.001 (*Figure 1*). In multivariable models for death or CV readmission at 2 years, there was a significant interaction between sex and 10-year age group (*P* < 0.001, Supplementary material online, *Table S4*).

For all secondary outcomes at 2 years, women had experienced significantly higher numbers of adverse events than men (CV readmission 26% vs. 21%, all-cause death 27% vs. 19%, CV death 14% vs. 10%, all *P* < 0.001).

### Characteristics and clinical outcomes by age group categories

Sex differences were further investigated within 10-year age groups. *Table 2* summarizes patient characteristics stratified by age group, ranging from 18 to 44 years up to ≥85 years at the time of admission for the index ACS event. Across increasing age groups, there were increasing proportions observed for women, European ethnicity, type of ACS as NSTEMI or MI unspecified, people residing in the two least deprived quintiles, and history of atherosclerotic CVD (without ACS), atrial fibrillation, and heart failure.

**Table 2:** Clinical characteristics of men and women admitted with first-time acute coronary syndrome from 2010 to 2016 stratified by 10-year age groups

	18–44 years *n* = 2695 (4%)		45–54 years *n* = 8081 (13%)		55–64 years *n* = 12 912 (20%)		65–74 years *n* = 15 072 (24%)		75–84 years *n* = 14 562 (23%)		≥85 years *n* = 9923 (16%)	
	F *n* = 653 (24%)	M *n* = 2042 (76%)	F *n* = 2114 (26%)	M *n* = 5967 (74%)	F *n* = 3894 (30%)	M *n* = 9018 (70%)	F *n* = 5741 (38%)	M *n* = 9331 (62%)	F *n* = 7028 (48%)	M *n* = 7534 (52%)	F *n* = 6072 (61%)	M *n* = 3851 (39%)
Ethnicity, *n* (%)												
European	310 (48)	1037 (51)	1112 (53)	3704 (62)	2455 (63)	6441 (71)	4339 (76)	7470 (80)	6010 (86)	6496 (86)	5617 (93)	3560 (92)
Māori	220 (34)	391 (19)	607 (29)	1019 (17)	826 (21)	1115 (12)	705 (12)	763 (8)	440 (6)	391 (5)	107 (2)	61 (2)
Pacific peoples	78 (12)	264 (13)	218 (10)	526 (9)	315 (8)	542 (6)	307 (5)	426 (5)	210 (3)	227 (3)	94 (2)	53 (1)
Indian	23 (4)	206 (10)	104 (5)	415 (7)	148 (4)	484 (5)	187 (3)	311 (3)	113 (2)	137 (2)	45 (1)	32 (1)
Chinese/other Asian	14 (2)	102 (5)	55 (3)	202 (3)	117 (3)	285 (3)	140 (2)	230 (3)	159 (2)	169 (2)	87 (1)	59 (2)
Other	8 (1)	42 (2)	18 (1)	101 (2)	33 (1)	151 (2)	63 (1)	131 (1)	96 (1)	114 (2)	122 (2)	86 (2)
History of atherosclerotic CVD^a^	78 (12)	112 (6)	258 (12)	593 (10)	684 (18)	1471 (16)	1414 (25)	2485 (27)	2278 (32)	2831 (38)	2205 (36)	1599 (42)
Prior PCI or CABG	6 (1)	17 (1)	32 (2)	136 (2)	107 (3)	344 (4)	210 (4)	657 (7)	257 (4)	663 (9)	128 (2)	231 (6)
History of PVD	22 (3)	15 (1)	67 (3)	106 (2)	162 (4)	289 (3)	397 (7)	630 (7)	601 (9)	914 (12)	544 (9)	498 (13)
History of AF	14 (2)	38 (2)	69 (3)	155 (3)	183 (5)	452 (5)	553 (10)	884 (10)	1184 (17)	1323 (18)	1406 (23)	913 (24)
History of heart failure	26 (4)	65 (3)	90 (4)	136 (2)	176 (5)	303 (3)	453 (8)	499 (5)	913 (13)	842 (11)	1213 (20)	723 (19)
M3 index, mean (SD)	0.29 (0.62)	0.14 (0.42)	0.29 (0.62)	0.15 (0.44)	0.31 (0.63)	0.22 (0.54)	0.44 (0.75)	0.35 (0.68)	0.54 (0.77)	0.54 (0.79)	0.59 (0.73)	0.63 (0.77)
NZ deprivation index (quintile), *n* (%)												
1 (least deprived)	81 (12)	315 (15)	237 (11)	899 (15)	493 (13)	1461 (16)	709 (12)	1520 (16)	887 (13)	1097 (15)	849 (14)	588 (15)
2	91 (14)	299 (15)	287 (14)	1034 (17)	597 (15)	1483 (16)	964 (17)	1646 (18)	1269 (18)	1346 (18)	1163 (19)	723 (19)
3	104 (16)	354 (17)	330 (16)	1050 (18)	696 (18)	1657 (18)	1121 (20)	1810 (19)	1428 (20)	1606 (21)	1361 (22)	864 (22)
4	137 (21)	437 (21)	478 (23)	1263 (21)	855 (22)	1997 (22)	1422 (25)	2130 (23)	1836 (26)	1842 (24)	1614 (27)	970 (25)
5 (most deprived)	239 (37)	616 (30)	757 (36)	1649 (28)	1190 (31	2244 (25)	1434 (25)	2065 (22)	1544 (22)	1549 (21)	1059 (17)	677 (18)
NZ Deprivation index missing	1 (0.2)	21 (1)	25 (1)	72 (1)	63 (2)	176 (2)	91 (2)	160 (2)	64 (1)	94 (1)	26 (0.4)	29 (1)
Type of ACS												
STEMI	169 (26)	779 (38)	448 (21)	2014 (34)	798 (21)	2582 (29)	1085 (19)	2091 (22)	1131 (16)	1274 (17)	884 (15)	495 (13)
NSTEMI	335 (51)	933 (46)	1095 (52)	2825 (47)	2070 (53)	4458 (49)	3152 (55)	4888 (52)	4261 (61)	4495 (60)	3999 (66)	2547 (66)
UA	122 (19)	284 (14)	510 (24)	975 (16)	899 (23)	1725 (19)	1257 (22)	1932 (21)	1129 (16)	1273 (17)	563 (9)	376 (10)
MI unspecified	27 (4)	46 (2)	61 (3)	153 (3)	253 (3)	380 (3)	247 (4)	420 (5)	507 (7)	492 (7)	626 (10)	433 (11)
**In-hospital mortality**	20 (3)	41 (2)	43 (2)	123 (2)	134 (3)	286 (3)	331 (6)	547 (6)	771 (11)	830 (11)	1142 (19)	786 (20)
**30 days** ^b^												
Composite: all-cause death/CV readmission	34 (5)	81 (4)	101 (5)	239 (4)	201 (5)	428 (5)	394 (7)	557 (6)	734 (12)	721 (11)	862 (18)	572 (19)
CV readmission^c^	32 (5)	78 (3)	91 (4)	219 (4)	181 (5)	370 (4)	329 (6)	438 (5)	557 (9)	532 (8)	579 (12)	368 (12)
All-cause death	3 (1)	6 (0.5)	12 (1)	29 (0.5)	28 (1)	75 (1)	91 (2)	139 (2)	220 (4)	249 (4)	375 (8)	268 (9)
CV death	1 (0.5)	3 (0.5)	5 (0.2)	16 (0.3)	7 (0.2)	38 (0.4)	34 (1)	66 (1)	108 (2)	122 (2)	231 (5)	150 (5)
**2 years** ^b^												
Composite: all-cause death/CV readmission	129 (20)	279 (14)	407 (20)	900 (16)	816 (22)	1733 (20)	1533 (28)	2315 (26)	2767 (44)	3037 (45)	3178 (65)	2138 (70)
CV readmission^b^	121 (19)	259 (13)	363 (18)	827 (14)	706 (19)	1493 (17)	1231 (23)	1812 (21)	2108 (34)	2239 (33)	2023 (41)	1376 (45)
All-cause death	19 (3)	43 (2)	95 (5)	147 (3)	221 (6)	439 (5)	604 (11)	913 (10)	1357 (22)	1609 (24)	2194 (45)	1532 (50)
CV death	6 (1)	18 (1)	35 (2)	71 (1)	66 (2)	171 (2)	194 (4)	351 (4)	606 (10)	751 (11)	1244 (25)	799 (26)

Values are *n* (column percentage), median (interquartile range), or mean (standard deviation).

ACS, acute coronary syndrome; AF, atrial fibrillation; CABG, coronary artery bypass graft; CVD, cardiovascular disease; NSTEMI, non-ST-elevation myocardial infarction; PCI, percutaneous coronary intervention; PVD, peripheral vascular disease; STEMI, ST-elevation myocardial infarction.

^a^includes admissions for atherosclerotic CVD excluding prior ACS.

^b^30-day, 2-year events and % exclude in-hospital events.

^c^Includes all CV readmissions not just those within the composite outcome (same with other secondary outcomes).

Within age groups, women had higher proportions of prior CVD hospitalizations than men in the younger age groups (up to 64 years), with the reverse in older age groups (*Table 2*). Sex differences in type of ACS also varied with age. While the proportion of patients with STEMI steadily decreased for both men and women, men decreased from 38% STEMI in those aged 18–44 years to 13% of those aged ≥85, and women changed from 25% STEMI for those 18–44 years to 15% of those aged ≥85.

Within 10-year age groups, there were no significant differences in the odds of in-hospital death between men and women, except for those aged ≥85 years where women were at marginally lower risk [OR for women = 0.90 (0.82–1.00), *P* = 0.049] (see Supplementary material online, *Table S5*), although in the multivariable model, this effect size was no longer statistically significant [adjusted OR = 0.91 (0.81–1.01), *P* = 0.067] (see Supplementary material online, *Table S6*).

**Figure 2:**
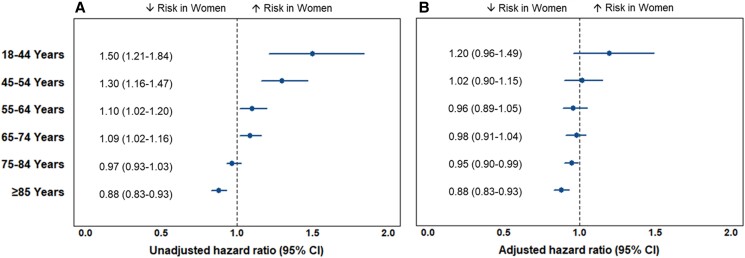
Hazard ratios (95% confidence interval) for women vs. men for the composite outcome of death or cardiovascular readmission at 2 years; separate models were performed in each age group. (*A*) Unadjusted hazard ratios. (*B*) Hazard ratios adjusted for the M3 multimorbidity index, type of acute coronary syndrome, ethnicity, and NZDep2013. CI, confidence interval.

In the unadjusted models for time to death or CV readmission, for all age groups <75 years, women were at higher risk of adverse events than men (*Figure 2A*). For people aged ≥85 years, this association reversed with women at lower risk than men [HR for women = 0.88 (0.83–0.93), *P* < 0.001]. In the multivariable models, there were no sex differences up to age <75 years, and women aged ≥75 years were at lower risk of events than men [adjusted HR for 75–84 years = 0.95 (0.90–0.99), *P* = 0.049, and for ≥85 years = 0.88 (0.83–0.93), *P* < 0.001] (*Figure 2B*; Supplementary material online, *Table S7*).

The relative importance of the predictors within each age group-specific model also changed across age groups. While the comorbidity burden (M3 index) was a significant predictor across all age groups, it was particularly important in younger patients (see Supplementary material online, *Table S7*). In contrast, type of ACS was not a significant predictor in younger age groups but was for those aged over 55. The influence of ethnicity on the risk of death or CV readmission within each model also varied by age group, with patients of Māori ethnicity at the highest risk of adverse events compared with Europeans in those aged under 75 years, and patients of other ethnicities at the highest risk for those aged over 75 years (see Supplementary material online, *Table S7*).

A similar pattern of higher unadjusted risk for women in younger age groups was also seen for the secondary outcomes (all-cause death, CV death, and CV readmission); however, this was only statistically significant for the women aged <55 years for CV readmission and for women aged 45–54 for all-cause death (see Supplementary material online, *Figures S1–S3*).

## Discussion

Among over 60 000 people admitted for a first-time ACS in NZ, we have shown that women are on average 7 years older at the time of admission and overall have a higher risk of experiencing both short- and long-term adverse events compared with men. Differing age and comorbidity profiles for men and women result in contrasting age-specific sex differences in risk between the youngest and oldest age groups.

Although women were more likely to die during the index ACS admission, multivariable adjustment amongst the whole cohort changed the direction of the OR to indicate women were at lower risk than men, highlighting the importance of age, multimorbidity, deprivation levels, type of ACS, and ethnicity to in-hospital mortality risk relative to the importance of sex. While many studies have assessed sex differences in in-hospital mortality after ACS or MI, mostly showing higher unadjusted rates for women that are attenuated or eliminated by adjustment for age and comorbidities,^8–11,24^ age-specific analyses allow more nuance.^25^ In a recent report of almost 7 million people admitted with acute MI in the USA, the adjusted odds of dying in hospital were age specific with younger and middle-aged women at higher risk than men and older women at lower risk than men.^26^

The type of ACS was a consistent predictor of in-hospital death across all age groups. Of note, those with ‘MI unspecified’ were at much higher odds of in-hospital death compared with those with NSTEMI, ranging from 30 times greater in those aged 18–44 to 7 times greater in those aged over 85. This relatively small heterogeneous group of patients (5% overall) represents a mixture of type 1 and 2 MI, including type 1 that cannot be further categorized (including late presentations and those too unwell for investigations to be completed) and type 2 with other acute diagnoses, including critically unwell non-cardiac diagnoses.

In this study, women aged under 65 were at a higher unadjusted risk of 2-year death or CV readmission than men. As seen for in-patient mortality, this was no longer significant after multivariable adjustment, indicating 2-year risk in younger women can also be accounted for by differences in comorbidities, type of ACS, ethnicity, and the NZ deprivation index. These findings are consistent with other age-stratified studies where younger women were also at higher risk of death or hospital readmission after discharge, particularly for young women with STEMI compared with young men with STEMI.^12,16–19^ Amongst 29 000 patients admitted with ACS in the Netherlands, analysis of 1-year mortality showed a significant age-sex interaction with higher mortality in women up until age 71 and lower mortality at advanced age.^13^ Similarly, amongst 15 500 patients with STEMI enrolled in seven Arabian Gulf registries, women had higher 1-year mortality compared with men, with the difference in risk diminishing with age.^15^

Several reasons for worse outcomes in younger women following ACS have been proposed. Younger women experiencing ACS have been shown to have a different risk factor profile to younger men,^14^ and in the current study, young women had a higher burden of comorbidities and prior hospitalizations for atherosclerotic CVD, PVD, and heart failure than young men. In addition, young women were also more often of Māori ethnicity and more likely to be living in areas of highest socio-economic deprivation than young men. We have previously shown that amongst NZ patients aged <55 years undergoing angiography for first-time ACS, young women were more likely than young men to be of Māori ethnicity, to have diabetes and to have a BMI of ≥30 kg/m^2^, though men and women had equally high rates of current smoking (48%).^27^

There are consistent data showing women experience delays in presentation to hospital and lower use of guideline-recommended acute treatments during an ACS admission, as well as lower use of secondary prevention therapies, with differences not fully explained by age and comorbidities.^28–30^ In addition, the randomized clinical trials underlying clinical guidelines for patients with ACS do not fully represent the diversity of the ACS population, including under-representation of women and the elderly.^31,32^

As well as potential bias, differences in the use of and response to therapy may be influenced by biological differences and uncertainties in diagnosis. For example, women are more likely to present with MI with non-obstructive coronary arteries (MINOCA), and amongst 8305 NZ patients with MI undergoing angiography in a previous study, 10.8% had MINOCA—19.3% of the women and 7.1% of the men—with these patients less likely to receive evidence-based treatments than those with obstructive coronary disease.^33^ Recent papers argue MINOCA should be considered a working diagnosis, with variable underlying mechanisms that require further clinical investigation.^34^

For older patients in our study, men were at higher risk of adverse events post-discharge than women (>85 years for the unadjusted and >75 years in the adjusted models), driven primarily by mortality rather than CV readmission. While a decreased risk for older men compared with older women has been reported previously,^13,35,36^ this has not been a consistent finding in age-stratified studies.^12,16,17^ Possible reasons for these findings include a longer lifetime exposure to coronary artery disease in older men compared with older women and a shorter overall life expectancy.

Men and women may also differ in the types of adverse events experienced after an ACS event. Other research on long-term outcomes post-MI has shown similar CV mortality, but higher all-cause mortality, in young women compared with young men (aged ≤50 years).^37^ Similarly, for hospital readmissions, sex differences were greatest for non-cardiac rehospitalizations after acute MI in the VIRGO study, possibly due to the greater comorbidity and socio-economic burdens of young women.^19^ In our study, there were sex differences in all-cause but not CV mortality at 2 years; however, with a low number of CV deaths in younger women, examination of longer-term outcomes (including non-cardiac and repeated admissions) for these patients will be important to understand the full burden of disease.

### Limitations

The national datasets used in this study rely on ICD-10-coded data meaning detailed information on ACS presentation, CV risk factors, intervention, and other in-hospital management was not available for this study. Given the importance of revascularization on outcomes, post-ACS, and the potential for unequal use of revascularization in men and women, our inability to adjust for this may have impacted on our results. The All New Zealand Acute Coronary Syndrome Quality Improvement (ANZACS-QI) registry includes all patients undergoing angiography in NZ and collects extensive clinical, laboratory, in-hospital management, and angiographic data.^38^ Subsequent research will further investigate sex differences in those patients enrolled in ANZACS-QI registry, including the effect of management and subsequent medical treatment on differences in clinical outcomes.

## Conclusion

Sex differences in adverse events following first-time ACS are influenced by varying age and comorbidity profiles for women and men. Younger women have a higher risk of death or CV readmission than younger men, largely due to differences in comorbidities, type of ACS, ethnicity, and deprivation levels.

## Supplementary material

Supplementary material is available at *European Heart Journal: Acute Cardiovascular Care* online.

## Supplementary Material

zuad151_Supplementary_Data

## Data Availability

Data are available upon reasonable request. All individual participant (de-identified) data, including a data dictionary defining each field, will be made available to university based academic researchers if their proposed analyses are approved by the investigators’ data access proposal committee. A proposal must be considered relevant to the original aims of the research, must meet the study's ethics approval criteria, and will require one or more of the study investigators as formal collaborators. A signed data access agreement will be required and the costs of preparing the datasets will need to be covered. There are no set dates for when these data will be made available. Please contact the corresponding author regarding data sharing requests.
