# Supplementary material for: Sex differences in outcomes after acute coronary syndrome vary with age: a New Zealand national study
Source: Eur Heart J Acute Cardiovasc Care. 2023 Dec 12;13(3):284–92. doi: 10.1093/ehjacc/zuad151 (PMC10927026; doi:10.1093/ehjacc/zuad151)
Supplement: zuad151_Supplementary_Data [file zuad151_supplementary_data.docx]

**Supplementary Data**

**Supplementary Table 1. ICD-10-AM and ACHI codes used to define a cardiovascular readmission**

| **Outcome type** | **ICD-10-AM codes and ACHI procedure codes** |
| --- | --- |
| Myocardial infarction | I210, I211 - I214, I219 - I221, I228, I229 |
| Unstable angina | I200 |
| Other ischaemic heart disease | I201, I208, I209, I230 - I236, I238, I240, I248, I249, I253 - I256, I460, I461, I469 |
| Heart failure | I110, I130, I132, I500, I501, I509 |
| Coronary angioplasty/stents | 3530400, 3530500, 3531000-3531002, 3830000, 3830300, 3830600-3830602, 3830900, 3831200, 3831201, 3831500, 3831800, 3831801, 9021800-9021803, 3530401, 3530501, 3531003-3531005, 3830001, 3830301, 3830603-3830605, 3845619, 3850500, 3850700, 3850800, 3850900 |
| CABG | 3849700-3849707, 3850000-3850005,  3850300-3850305, 3863700, 3865002, 3865003, 3865308, 9020100-9020103 |
| Ischaemic stroke | I630 - I636, I638, I639, I64 |
| Haemorrhagic stroke | I600 - I616, I618, I619 |
| Transient ischaemic attack | G450 - G453, G458 - G459 |
| Other cerebrovascular disease | G460-G468, I651, I660-I664, I668-I670 |
| Peripheral vascular disease | E1050 - E1052, E1150 - E1152, E1350-1352, E1451, E1452, I650, I652, I653, I658, I659, I7021 - I7024, I7100 - I7103, I713, I715, I718, I739 - I745, I748, I749 |
| PVD procedures | 3270000-3270011, 3270300, 3270800-3270803,  3271200, 3271201, 3271500-3271503, 3271800,  3271801, 3272100, 3272101, 3272400, 3272401,  3273000, 3273001, 3273300, 3273301, 3273600,  3273900, 3274200, 3274500, 3274800,  3275100-3275103, 3275400-3275402, 3275700,  3275701, 3276300-3276303, 3276305-3276314, 3276316-3276319, 3305000, 3305500,  3307500, 3308000, 3310000, 3311200, 3311500, 3311800, 3312100, 3312400, 3312700, 3313000,  3313001, 3313900, 3314200, 3314800, 3315100,  3315400, 3315700, 3316000, 3316300, 3317200,  3317800, 3318100, 3350000, 3350600, 3350601,  3350900, 3351200, 3351500, 3351501, 3351800,  3352100, 3352400, 3352700, 3353000, 3353001,  3353300, 3353600, 3353900, 3354200,  3354800-3354803, 3530000, 3530301, 3530304,  3530306, 3530307, 3530600-3530602, 3530700,  3530701, 3530900-3530902, 3530906-3530909,  3531200, 3531201, 3531500, 3531501,  9021100-9021106, 9021200-9021210, 9022900,  9023000, 9023100 |
| Cardiac transplantation | 9020500, 9020501, Z994 |

* ICD=International Classification of Diseases; ICD-10-AM=ICD, 10^th^ Revision, Australian Modification; ACHI=Australian Classification of Health Interventions; CABG=Coronary artery bypass surgery; PVD=peripheral vascular disease.

**Supplementary Table 2*.* Logistic regression for in-hospital** **death, unadjusted.**

|  | **Odds Ratio (95%CI)** | **p** |
| --- | --- | --- |
| Men | Reference |  |
| Women | 1.42 (1.34-1.51) | <0.001 |

**Supplementary Table 3*.* Logistic regression for in-hospital** **death, multivariable adjusted.**

|  | **Odds Ratio (95%CI)** | **p** |
| --- | --- | --- |
| Sex |  |  |
| Men | Reference |  |
| Women | 0.94 (0.88-1.00) | 0.064 |
| Age (per year) | 1.07 (1.06-1.07) | <0.001 |
| M3 Multimorbidity index | 1.68 (1.62-1.74) | <0.001 |
| Type of ACS |  |  |
| NSTEMI | Reference |  |
| STEMI | 3.12 (2.89-3.37) | <0.001 |
| Unstable angina | 0.34 (0.29-0.39) | <0.001 |
| MI Unspecified | 9.70 (8.88-10.59) | <0.001 |
| Ethnicity |  |  |
| European | Reference |  |
| Māori | 1.38 (1.22-1.55) | <0.001 |
| Pacific peoples | 1.41 (1.21-1.64) | <0.001 |
| Indian | 1.05 (0.84-1.31) | 0.637 |
| Chinese/Other Asian | 1.15 (0.92-1.43) | 0.214 |
| Other | 2.67 (2.19-3.24) | <0.001 |
| NZ Deprivation index |  |  |
| 1 (least deprived) | Reference |  |
| 2 | 0.95 (0.84-1.07) | 0.369 |
| 3 | 1.22 (1.09-1.37) | <0.001 |
| 4 | 1.20 (1.08-1.34) | <0.001 |
| 5 (most deprived) | 1.41 (1.26-1.58) | <0.001 |

ACS=acute coronary syndrome; STEMI=ST-elevation myocardial infarction; NSTEMI= non-ST-elevation myocardial infarction.

**Supplementary Table 4*.* Cox proportional hazards models for time to death or CV readmission at 2 years. Adjusted for age group, the M3 multimorbidity index, type of ACS, ethnicity, the NZDep13 deprivation index and age group:sex interaction term.**

|  | **Odds Ratio (95%CI)** | **p** |
| --- | --- | --- |
| Sex |  |  |
| Men | Reference |  |
| Women | 0.99 (0.94-1.07) | 0.979 |
| Age group |  |  |
| <45 years | 0.52 (0.46-0.58) | <0.001 |
| 45-55 years | 0.58 (0.54-0.63) | <0.001 |
| 55-65 years | 0.75 (0.71-0.80) | <0.001 |
| 65-75 years | Reference |  |
| 75-85 years | 1.78 (1.68-1.88) | <0.001 |
| ≥85 years | 3.30 (3.11-3.50) | <0.001 |
| M3 Multimorbidity index | 1.68 (1.65-1.70) | <0.001 |
| Type of ACS |  |  |
| NSTEMI | Reference |  |
| STEMI | 0.90 (0.86-0.93) | <0.001 |
| Unstable angina | 0.88 (0.84-0.91) | <0.001 |
| MI Unspecified | 1.05 (0.98-1.12) | 0.158 |
| Ethnicity |  |  |
| European | Reference |  |
| Māori | 1.37 (1.31-1.44) | <0.001 |
| Pacific peoples | 1.17 (1.10-1.26) | <0.001 |
| Indian | 0.99 (0.91-1.09) | 0.956 |
| Chinese/Other Asian | 0.79 (0.69-0.86) | <0.001 |
| Other | 1.26 (1.13-1.41) | <0.001 |
| NZ Deprivation index |  |  |
| 1 (least deprived) | Reference |  |
| 2 | 1.06 (1.01-1.12) | 0.029 |
| 3 | 1.11 (1.05-1.16) | <0.001 |
| 4 | 1.16 (1.10-1.21) | <0.001 |
| 5 (most deprived) | 1.24 (1.18-1.30) | <0.001 |
| Age group: Sex interaction |  | <0.001 * |
| <45 years: Sex | 1.26 (1.01-1.57) |  |
| 45-55 years: Sex | 1.13 (0.99-1.29) |  |
| 55-65 years: Sex | 1.00 (0.90-1.11) |  |
| 65-75 years: Sex | Reference |  |
| 75-85 years: Sex | 0.95 (0.87-1.03) |  |
| ≥85 years: Sex | 0.89 (0.82-0.97) |  |

*test of overall interaction between 10y age group and sex

**Supplementary Table 5*.* Logistic regression for in-hospital** **death, unadjusted. Separate models were calculated for each age group.**

|  | **Odds ratio (95% CI)** | | | | | |
| --- | --- | --- | --- | --- | --- | --- |
|  | **18-44 years**  n=2695 | **45-54 years**  n=8081 | **55-64 years**  n=12912 | **65-74 years**  n=15072 | **75-84 years**  n=14562 | **≥85 years**  n=9923 |
| In-hospital death, n(%) | 61 (2) | 166 (2) | 420 (3) | 878 (6) | 1601 (11) | 1928 (19) |
| Sex |  |  |  |  |  |  |
| Men | Reference | Reference | Reference | Reference | Reference | Reference |
| Women | 1.54  (0.88-2.62)  p=0.117 | 0.99  (0.69-1.39)  p=0.939 | 1.09  (0.88-1.34)  p=0.428 | 0.98  (0.85-1.13)  p=0.806 | 0.99  (0.90-1.10)  p=0.929 | 0.90 (0.82-1.00)  p=0.049 |

**Supplementary Table 6*.* Logistic regression for in-hospital death, multivariable adjusted. Separate models were calculated for each age group.**

|  | **Odds ratio (95% CI)** | | | | | |
| --- | --- | --- | --- | --- | --- | --- |
|  | **18-44 years**  n=2613 | **45 - 54 years**  n=7822 | **55 - 64 years**  n=12,271 | **65 - 74 years**  n=13,969 | **75 - 84 years**  n=12,818 | **≥85 years**  n=7946 |
| In-hospital death, n (%) | 61 (2) | 166 (2) | 420 (3) | 878 (6) | 1601 (11) | 1928 (19) |
| Sex |  |  |  |  |  |  |
| Men | Ref | Ref | Ref | Ref | Ref | Ref |
| Women | 1.73 (0.94-3.12)  p=0.072 | 0.82 (0.55-1.22)  p=0.335 | 1.06 (0.84-1.32)  p=0.644 | 0.94 (0.80-1.09)  p=0.403 | 0.96 (0.86-1.08)  p=0.495 | 0.90 (0.81-1.01)  p=0.067 |
| M3 index | 1.74 (1.20-2.44) | 2.57 (2.13-3.08) | 2.03 (1.81-2.28) | 1.85 (1.72-2.00) | 1.59 (1.49-1.69) | 1.45 (1.35-1.55) |
| Type of ACS |  |  |  |  |  |  |
| NSTEMI | Ref | Ref | Ref | Ref | Ref | Ref |
| STEMI | 6.90 (3.39-15.60) | 4.86 (3.21-7.52) | 4.15 (3.25-5.34) | 2.64 (2.22-3.14) | 3.04 (2.65-3.48) | 3.17 (2.76-3.63) |
| Unstable angina | Inestimable* | 0.57 (0.23-1.22) | 0.27 (0.14-0.48) | 0.28 (0.19-0.39) | 0.31 (0.23-0.41) | 0.45 (0.34-0.58) |
| MI Unspecified | 30.01 (12.27-77.03) | 24.90 (14.98-41.68) | 15.87 (11.60-21.70) | 9.95 (8.11-12.21) | 10.20 (8.76-11.89) | 7.43 (6.45-8.58) |
| Ethnicity |  |  |  |  |  |  |
| European | Ref | Ref | Ref | Ref | Ref | Ref |
| Māori | 1.61 (0.75-3.42) | 1.18 (0.76-1.78) | 1.55 (1.17-2.03) | 1.30 (1.03-1.63) | 1.25 (1.00-1.56) | 0.86 (0.53-1.34) |
| Pacific peoples | 2.35 (1.03-5.22) | 1.41 (0.82-2.38) | 1.92 (1.33-2.74) | 1.33 (0.98-1.78) | 1.06 (0.77-1.42) | 0.99 (0.63-1.51) |
| Indian | 1.21 (0.28-3.76) | 1.15 (0.50-2.30) | 0.84 (0.45-1.43) | 1.63 (1.12-2.32) | 1.00 (0.63-1.51) | 0.53 (0.25-1.00) |
| Chinese/Other Asian | 4.54 (1.55-11.76) | 1.40 (0.42-3.49) | 1.52 (0.80-2.65) | 0.85 (0.47-1.42) | 1.25 (0.87-1.78) | 0.86 (0.53-1.34) |
| Other | 9.12 (2.43-27.85) | 2.63 (0.88-6.37) | 3.69 (1.90-6.60) | 3.00 (1.86-4.66) | 2.83 (1.95-2.04) | 1.95 (1.40-2.68) |
| NZ Deprivation index |  |  |  |  |  |  |
| 1 (least deprived) | Ref | Ref | Ref | Ref | Ref | Ref |
| 2 | 1.77 (0.56-6.15) | 0.87 (0.38-2.02) | 0.54 (0.35-0.84) | 1.13 (0.84-1.52) | 0.89 (0.72-1.11) | 1.03 (0.85-1.25) |
| 3 | 1.05 (0.32-3.75) | 1.56 (0.76-3.41) | 0.74 (0.51-1.07) | 1.44 (1.10-1.90) | 1.21 (0.99-1.49) | 1.22 (1.02-1.47) |
| 4 | 1.62 (0.57-5.32) | 2.06 (1.06-4.31) | 0.88 (0.63-1.26) | 1.36 (1.04-1.79) | 1.26 (1.04-1.54) | 1.09 (0.91-1.30) |
| 5 (most deprived) | 2.08 (0.78-6.64) | 2.13 (1.12-4.43) | 0.90 (0.64-1.28) | 1.57 (1.20-2.07) | 1.55 (1.27-1.89) | 1.24 (1.03-1.51) |

* Zero deaths in patients with unstable angina aged 18-44 years. ACS=acute coronary syndrome; STEMI=ST-elevation myocardial infarction; NSTEMI= non-ST-elevation myocardial infarction.

|  | **Hazard ratio (95% CI)** | | | | | |
| --- | --- | --- | --- | --- | --- | --- |
|  | **<45 years**  n=2613 | **45 - 54 years**  n=7822 | **55 - 64 years**  n=12,271 | **65 - 74 years**  n=13,969 | **75 - 84 years**  n=12,818 | **≥85 years**  n=7946 |
| Death or CV readmission, n (%) | 407 (16) | 1301 (17) | 2543 (21) | 3827 (27) | 5778 (45) | 5305 (67) |
| Sex |  |  |  |  |  |  |
| Men | Ref | Ref | Ref | Ref | Ref | Ref |
| Women | 1.19 (0.96-1.49)  p=0.111 | 1.02 (0.90-1.15)  p=0.736 | 0.96 (0.89-1.05)  p=0.386 | 0.98 (0.91-1.04)  p=0.449 | 0.95 (0.90-0.99)  p=0.049 | 0.88 (0.83-0.93)  p<0.001 |
| M3 Multimorbidity index | 2.22 (1.96-2.51) | 2.10 (1.96-2.24) | 2.02 (1.93-2.10) | 1.89 (1.84-1.95) | 1.59 (1.55-1.63) | 1.38 (1.33-1.43) |
| Type of ACS |  |  |  |  |  |  |
| NSTEMI | Ref | Ref | Ref | Ref | Ref | Ref |
| STEMI | 1.16 (0.93-1.45) | 1.04 (0.91-1.19) | 1.16 (1.06-1.27) | 0.84 (0.76-0.91) | 0.90 (0.84-0.98) | 0.89 (0.82-0.97) |
| Unstable angina | 1.07 (0.81-1.42) | 1.03 (0.89-1.19) | 0.96 (0.87-1.06) | 0.86 (0.79-0.91) | 0.79 (0.73-0.85) | 0.91 (0.83-0.99) |
| MI Unspecified | 1.15 (0.64-2.07) | 1.07 (0.77-1.48) | 2.05 (1.74-2.40) | 1.13 (0.97-1.33) | 1.10 (1.00-1.22) | 0.97 (0.86-1.08) |
| Ethnicity |  |  |  |  |  |  |
| European | Ref | Ref | Ref | Ref | Ref | Ref |
| Māori | 1.33 (1.04-1.71) | 1.40 (1.21-1.60) | 1.42 (1.28-1.58) | 1.31 (1.19-1.44) | 1.14 (1.02-1.26) | 1.20 (0.98-1.46) |
| Pacific peoples | 0.99 (0.72-1.36) | 1.05 (0.87-1.28) | 1.28 (1.11-1.49) | 1.18 (1.03-1.36) | 1.07 (0.93-1.24) | 1.12 (0.90-1.39) |
| Indian | 1.28 (0.89-1.85) | 0.85 (0.66-1.11) | 1.10 (0.91-1.33) | 1.07 (0.89-1.28) | 0.97 (0.79-1.20) | 0.77 (0.55-1.07) |
| Chinese/Other Asian | 0.44 (0.19-0.99) | 0.70 (0.46-1.07) | 0.83 ( 0.64-1.07) | 0.75 (0.59-0.94) | 0.70 (0.57-0.85) | 0.93 (0.74-1.17) |
| Other | 1.29 (0.61-2.76) | 1.27 (0.81-1.98) | 1.33 (0.95-1.86) | 1.20 (0.92-1.57) | 1.27 (1.03-1.58) | 1.22 (1.01-1.48) |
| NZ Deprivation index |  |  |  |  |  |  |
| 1 (least deprived) | Ref | Ref | Ref | Ref | Ref | Ref |
| 2 | 0.86 (0.56-1.32) | 1.07 (0.85-1.35) | 1.00 (0.85-1.17) | 1.15 (1.02-1.30) | 1.11 (1.01-1.22) | 0.97 (0.88-1.06) |
| 3 | 1.35 (0.93-1.97) | 1.16 (0.93-1.45) | 1.18 (1.02-1.37) | 1.18 (1.04-1.32) | 1.08 (0.99-1.19) | 1.01 (0.92-1.10) |
| 4 | 1.41 (0.99-2.03) | 1.30 (1.05-1.60) | 1.24 (1.08-1.43) | 1.26 (1.13-1.41) | 1.10 (1.00-1.20) | 1.05 (0.96-1.14) |
| 5 (most deprived) | 1.19 (0.83-1.70) | 1.49 (1.21-1.82) | 1.33 (1.16-1.53) | 1.35 (1.20-1.51) | 1.21 (1.10-1.32) | 1.05 (0.95-1.16) |

**Supplementary Table 7. Cox proportional hazards models for time to death or CV readmission at 2 years. Separate models were calculated for each age group, adjusted for the M3 multimorbidity index, type of ACS, ethnicity, and the NZDep13 deprivation index.**

ACS=acute coronary syndrome; STEMI=ST-elevation myocardial infarction; NSTEMI= non-ST-elevation myocardial infarction.

***Supplementary Figure 1.* Hazard ratios (95% CI) for women vs men for cardiovascular death at 2 years; separate models were performed in each age group. A: Unadjusted hazard ratios. B: Hazard ratios adjusted for the M3 multimorbidity index, type of ACS, ethnicity, and NZDep2013.**

***Supplementary Figure 2.* Hazard ratios (95% CI) for women vs men for all-cause death at 2 years; separate models were performed in each age group. A: Unadjusted hazard ratios. B: Hazard ratios adjusted for the M3 multimorbidity index, type of ACS, ethnicity, and NZDep2013.**

***Supplementary Figure 3.* Hazard ratios (95% CI) for women vs men for cardiovascular readmission at 2 years; separate models were performed in each age group. A: Unadjusted hazard ratios. B: Hazard ratios adjusted for the M3 multimorbidity index, type of ACS, ethnicity, and NZDep2013.**
